# Innate lymphoid cell regulation of adaptive immunity

**DOI:** 10.1111/imm.12639

**Published:** 2016-08-16

**Authors:** David R. Withers

**Affiliations:** ^1^ Institute of Immunology & ImmunotherapyCollege of Medical and Dental SciencesUniversity of BirminghamBirminghamUK

**Keywords:** innate lymphoid cells, memory, spleen and lymph nodes, T cells

## Abstract

Innate lymphoid cells (ILCs) were identified principally as non‐T‐cell sources of key cytokines, able to provide rapid and early production of these molecules in the support of tissue homeostasis, repair and response to infection. As our understanding of these cells has developed, it has become evident that ILCs can impact on lymphocytes through a range of mechanisms. Hence, an exciting area of research has evolved in determining the extent to which ILCs may regulate adaptive immune responses. This review will focus initially on our current understanding of where ILC populations are located and what this means for potential cellular interactions. Mechanisms underpinning such interactions and how they may contribute to controlling adaptive immunity will then be considered.

**Abbreviations**
APCs: antigen‐presenting cells
DCs: dendritic cells
GATA‐3: GATA‐binding protein 3
ILCs: innate lymphoid cells
IL: interleukin
NK: natural killer
ROR: retinoic‐acid‐receptor‐related orphan receptor
T‐bet: T‐box factor expressed in T cells
Th: T helper

## Introduction

Cells of the innate lymphoid cell (ILC) family have emerged in recent years as important players in the maintenance of tissue defence, repair and homeostasis, particularly at mucosal sites.^1^ Since their formal description in 2013,^2^ enormous progress has been made in characterizing these cells in both mice and humans and in understanding their developmental requirements. We now know a lot of what they can do; however, determining their contribution to immune responses *in vivo*, as part of an intact immune system, remains a substantial challenge. Multiple lines of evidence indicate important roles for these cells in the regulation of adaptive responses, particularly those of CD4^+^ T cells.^3^, ^4^, ^5^, ^6^ Yet fundamental questions remain concerning ILC contributions *in vivo* to adaptive immunity, notably regarding the precise mechanisms involved, but also where key cellular interactions occur and at what stage in the response. Progress has been limited by the lack of suitable *in vivo* models available to pinpoint ILC roles. With the emergence of superior approaches to test ILC functions,^4^ substantial headway in this area of research should be anticipated. Beyond an initial overview of ILC biology (many excellent reviews have been recently published including refs 1, 7, 8), here I will review the current understanding of ILC location within tissues, how this relates to their cellular interactions, and mechanisms through which ILCs may contribute to initiating, sustaining and even limiting adaptive immune responses.

## Overview of ILC groups

Innate lymphoid cells were defined as cells derived from a common lymphoid progenitor that are lymphoid in morphology but distinct from B and T cells, as they do not depend upon recombination activation gene‐mediated gene segment rearrangement to develop.^2^ Based upon the cytokines produced and the transcription factors controlling their development, three distinct groups were described mirroring several of the known effector CD4^+^ T‐cell subsets. Hence the group 1 ILCs (ILC1) contain those cells able to produce the T helper type 1 (Th1) cell‐associated cytokines interferon‐*γ* and tumour necrosis factor *α* and are at least partially dependent upon the transcription factor T‐box factor expressed in T cells (T‐bet).^9^ One major lineage within the ILC1 group are natural killer (NK) cells, recognized for many years as key cells in responding to viral infection and tumour surveillance.^10^ Although NK cells can be further split into several subsets, a second lineage distinct from NK cells does not require or express the transcription factor Eomesodermin, a close homologue of T‐bet.^9^ These Eomesodermin‐negative ILC1 appear to respond to intracellular infections.^11^, ^12^ The group 2 ILCs (ILC2) respond to signals including interleukin 25 (IL‐25), IL‐33 and thymic stromal lymphopoietin to produce some or all of the Th2‐associated cytokines IL‐4, IL‐5, IL‐9, IL‐13, so promoting not only anti‐helminth responses but also allergic inflammation.^13^, ^14^, ^15^, ^16^, ^17^ At least two subsets of ILC2 have been described to date.^18^ Although ILC2 were first recognized as being GATA‐binding protein 3 (GATA‐3) dependent,^19^ akin to Th2 cells,^20^ all ILC populations with the exception of conventional NK cells require GATA‐3 for their development.^21^, ^22^ GATA‐3 expression is maintained at high levels in ILC2, unlike other ILC populations, and ILC2 remain dependent upon continued GATA‐3 expression for their function.^19^ ILC2 development is also dependent upon the transcription factors retinoic‐acid‐receptor‐related orphan receptor (ROR)*α*,^23^ Bcl11b^24^ and ETS‐1.^25^

The third group of ILCs (ILC3) were defined as those cells able to produce the cytokines IL‐17A and IL‐22 and dependent upon the transcription factor ROR*γ*t for their development.^26^, ^27^, ^28^, ^29^ ILC3s are diverse in terms of known functions, including lymphoid organogenesis, antibacterial immunity and epithelial barrier protection.^26^, ^30^, ^31^, ^32^ In the adult, several phenotypically distinct ROR*γ*t‐expressing ILC populations have been described that may all contribute to mucosal barrier integrity through production of IL‐22 and Csf‐2 (granulocyte–macrophage colony‐stimulating factor), principally in response to IL‐23 and augmented by IL‐1*β* and TL1a.^32^, ^33^, ^34^ Despite being expressed at low levels relative to ILC2, continued GATA‐3 expression is required for at least some ILC3 functions.^35^ Surprisingly, the group‐defining transcription factor ROR*γ*t may not be required for several important ILC3 functions, in contrast to Th17 cells.^36^

Innate lymphoid cells are derived from a common ILC precursor,^8^ further differentiated than common lymphoid progenitors (such that it cannot give rise to B and T cells) and identified through its high expression of CD127 and *α*_4_ *β*_7_.^11^, ^37^, ^38^ The common ILC precursor differentiates into further precursor populations that retain the ability to form some ILC populations but not others.^37^

## ILC microenvironments

Understanding the location of ILCs within tissue and the potential cellular interactions of these cells is essential for understanding the *in vivo* functions of these cells. Through flow cytometric approaches ILCs have been described in a range of tissues; however, there are few precise details of their positioning with regard to other cell types. Dynamic *in vivo* imaging of ILC populations remains even more scarce,^39^ so much of our understanding of ILC location and their cellular interactions reflect a limited number of immunofluorescence snap shots.^26^, ^40^, ^41^ Using flow cytometry, ILCs are identified through an extensive panel of antibodies, so their identification by standard immunofluorescent techniques is challenging. This has limited identification of some populations such as Eomesodermin‐negative ILC1. ILC3 populations have been most studied, aided by tools that enable robust detection of ROR*γ*t.^26^, ^40^, ^42^, ^43^ To date, such experiments show that ILC populations are not evenly distributed throughout tissue, rather they are concentrated in specific regions, which one must assume relates to their functions.

### Secondary lymphoid tissues

**Figure 1:**
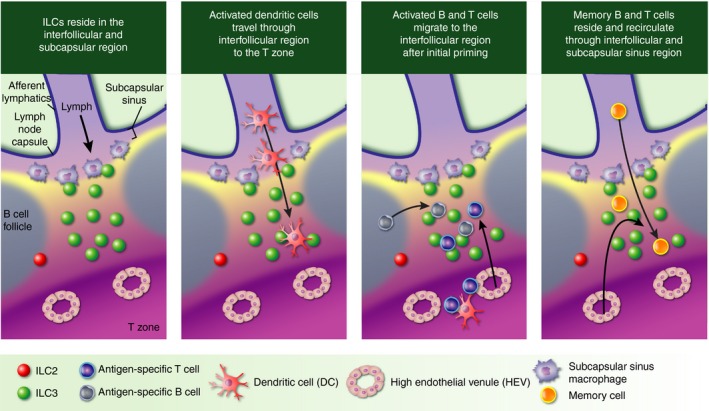
Innate lymphoid cells from groups 2 (ILC2) and 3 (ILC3) reside at key sites of lymphocyte traffic in secondary lymphoid tissue. Cartoon showing the location of ILC2 and ILC3 populations in lymph nodes^40^, ^41^ where both populations reside in the interfollicular spaces and at the interface of the B‐cell and T‐cell zones. This location facilitates potential interactions with (1) subscapular sinus macrophages located in the immediate vicinity; (2) activated dendritic cells (DCs) entering through the afferent lymph; (3) activated lymphocytes migrating to this region; (4) memory cells recirculating through the tissue.

When considering ILC contributions to adaptive immunity, an obvious point is that these cells have been described within all secondary lymphoid tissues, the key sites for initiating adaptive immune responses, particularly those of B and CD4^+^ T cells.^44^ However, the fundamental question remains – what do they actually do in these tissues? Compared with mucosal barrier tissues such as the intestine, the frequency of ILCs relative to other haematopoietic cells is very low in secondary lymphoid tissue. Of course, the vast majority of CD45^+^ cells in lymph nodes and spleen are naive lymphocytes recirculating through the tissue in search of cognate antigen, hence all non‐lymphocyte populations are rare in such tissues. Immunofluorescent studies showed that ILC3 (defined as ROR*γ*t^+^ CD3^−^ cells) are only present within a very distinct region of lymph nodes, the interfollicular region and interface between the B‐cell and T‐cell zones.^40^, ^41^ Evidence to date also indicate that ILC2 reside in this region of lymph nodes.^41^ This specific location is noteworthy because it is a key region through which lymphocytes, as well as other immune cells, traffic during different stages of adaptive responses.^45^, ^46^ Hence by residing here, ILC3 clearly have the potential to impact on multiple stages of B‐cell and T‐cell responses. As outlined in Fig. 1, activated dendritic cells (DCs) entering the lymph node via the afferent lymphatics pass through the interfollicular spaces on their way to the T zone.^45^ Activated B and CD4^+^ T cells specifically move to the interfollicular region during the early stages of the response and key differentiation events appear to be initiated here.^46^ Memory B‐ and T‐cell populations recirculate through secondary lymphoid tissue and also probably pass through this region.^47^, ^48^, ^49^

In other murine secondary lymphoid tissues, ILC3 are located within analogous locations of cellular trafficking, for example the marginal zone bridging channels of the splenic white pulp and the edge of the B‐cell follicles in Peyer's patches.^38^, ^40^, ^50^ Furthermore, in human spleen and lymph nodes, ILC3 also appear to be located in comparable locations.^51^, ^52^ Hence, although rare, ILCs appear to reside specifically within regions of secondary lymphoid tissue suggestive of common functions in B‐cell and T‐cell responses. Although in most tissues ILCs appear to be excluded from both the inner T zone and B‐cell follicle environments, an exception to this is in the isolated lymphoid follicles of the gut, where ILC3 are clearly located throughout the follicle and contribute to T‐cell‐independent switching to IgA via lymphotoxin *α*_1_ *β*_2_.^53^, ^54^ Among ILC1 populations, NK cells have been described in the splenic red pulp and T zone of lymph nodes although their location can change in response to infection.^55^, ^56^ Thus, although ILC2 and ILC3 may occupy similar locations within secondary lymphoid tissue, to date, members of the ILC1 group have been detected in different regions of this tissue.

### ILCs in ‘non‐lymphoid’ tissues

The positioning of ILCs in secondary lymphoid tissue is suggestive of roles in adaptive immune responses, but ILCs are clearly both more numerous and more frequent relative to other haematopoietic cells in non‐lymphoid tissues, particularly within mucosal barriers such as the lung and intestine, but also in tissues such as skin and fat. Imaging of cells in such tissues is often significantly more challenging than in secondary lymphoid tissue, and clear data on where ILC populations reside remains patchy. Many tissues described as ‘non‐lymphoid’ contain organized patches of lymphoid cells, best characterized in the intestine,^57^ but also present at other sites, for example in adipose tissue as fat‐associated lymphoid clusters.^14^, ^58^ Subgroups of ILCs may reside at different locations, facilitating specific functions. For example, ILC3 populations associated with lymphoid tissues are almost all NKp46^−^ MHCII^+^ whereas NKp46^+^ MHCII^−^ ILC3 appear to reside outside gut associated lymphoid tissues in the small intestine lamina propria.^42^ Within the lung and skin, it appears that ILCs are scattered within the tissue and probably only become concentrated in distinct sites after insult or inflammation.^6^, ^59^, ^60^ It remains to be clearly demonstrated whether ILC populations in adipose tissue are mostly within fat‐associated lymphoid clusters or located outside these structures.

What is the relationship between ILC populations in tissue and the secondary lymphoid tissues that drain these sites? Studies using parabiotic mice concluded that ILC are tissue‐resident cells that are maintained and expanded locally.^61^ Differentiation to ILC precursor populations occurs in the fetal liver and bone marrow,^37^, ^62^ before these cells seed peripheral tissues, and most appear to complete their maturation at these sites.^62^, ^63^, ^64^ Recent experiments using photoconvertible mice offer some evidence that ILC populations within lymph nodes may be supplemented by ILCs trafficking from local tissue.^41^ In summary, current evidence indicates that the majority of ILCs reside within non‐lymphoid tissues; however, clear populations exist in lymphoid tissues. Questions remain concerning the extent to which those ILCs in secondary lymphoid tissues are populated by cells trafficking from local tissues. Having drained to these sites, can ILCs then re‐enter the circulation? Based on parabiosis studies, if this occurs it involves only small numbers of ILCs.^61^ The specific functions that might be served by these small migratory ILC populations are currently unclear.

## How can ILCs regulate adaptive immune responses?

**Figure 2:**
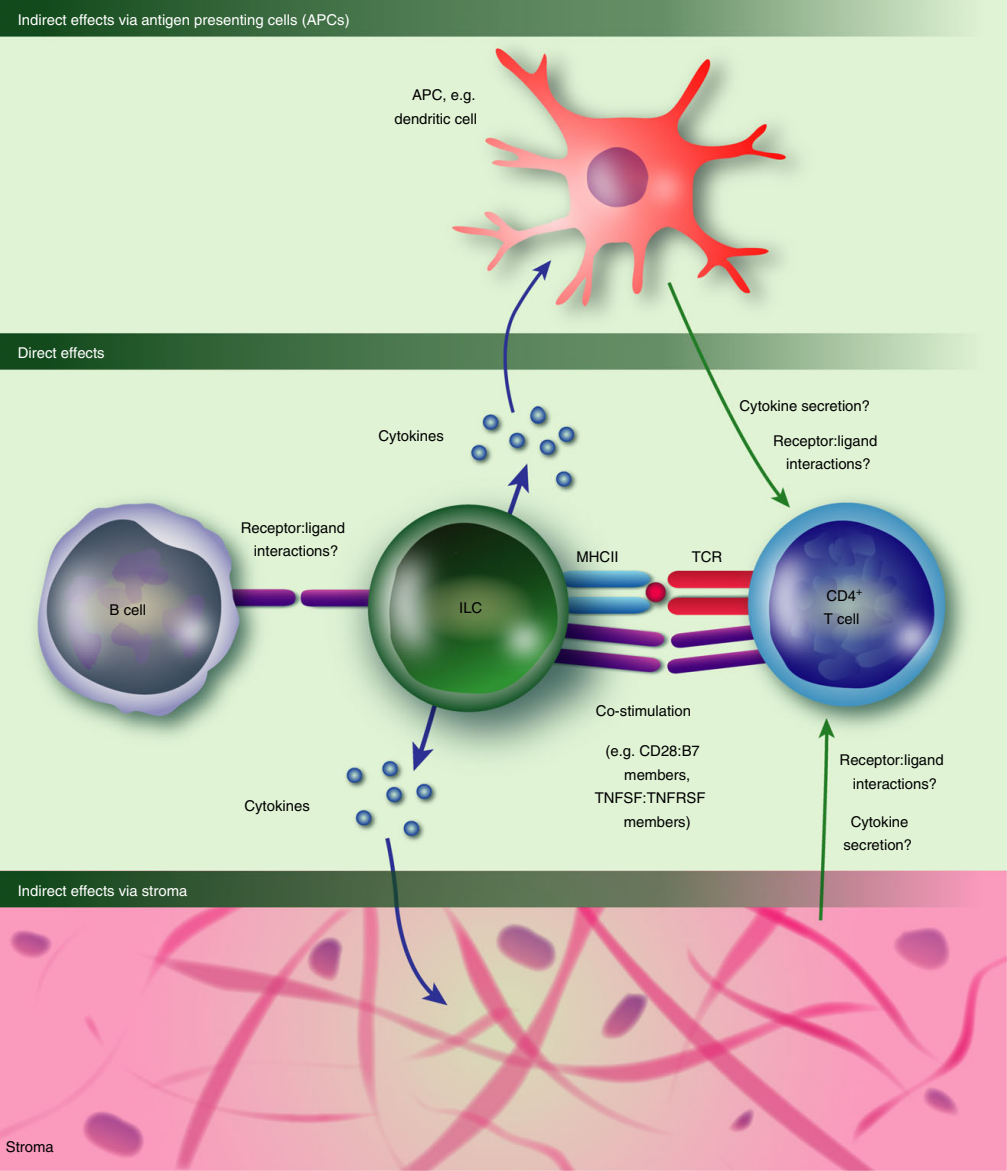
Mechanisms through which innate lymphoid cells (ILCs) may regulate adaptive immune responses. Cartoon showing how ILC populations may interact with lymphocytes, through both direct receptor–ligand interactions [including co‐stimulatory molecules such as B7 and tumour necrosis factor superfamily (TNFSF) members], as well as indirect effects through cytokines affecting stroma and other classical antigen‐presenting cells (APCs) in the local environment.

In discussing how ILC populations impact on adaptive immune responses, it is clear that many questions remain in our understanding of their precise roles. This reflects the limitations of current *in vivo* models and the relatively few investigations in this area. Here I will review how ILC populations may affect adaptive immune responses, focusing on specific examples where robust data exist. Simplistically, the roles identified to date can be split into indirect effects on lymphocytes mediated by other cells types and direct interactions with B and T cells. These possible interactions are summarized in Fig. 2.

### ILCs: middle men in regulating adaptive immune responses?

Innate lymphoid cell cytokine production clearly contributes to regulating tissue protection through innate mechanisms.^12^, ^65^ Given their rapid and substantial production of some cytokines, ILCs also probably contribute to driving T helper cell differentiation. For example NK cell production of interferon‐*γ* may contribute towards Th1 differentiation, ILC2 appear to be a critical source of IL‐4 in driving Th2 differentiation following helminth infection.^66^, ^67^ Where further progress has been made, is in understanding how ILCs can influence other local innate populations that then impact on the success of the lymphocyte response. Hence, recent evidence indicates the ILCs serve as middle‐men, responding to signals in the tissue and helping to orchestrate progression of the adaptive response, both at the site of insult, and in the draining secondary lymphoid tissue. Within the lung, it is now evident that ILC2 impact on several stages of the Th2 CD4^+^ T‐cell response to allergens through interactions with DC populations. In establishing the initial response, ILC2‐derived IL‐13 is required for activated DCs homing to the draining lymph nodes and hence for efficient Th2 cell priming.^3^ After antigen re‐encounter, ILC2‐derived IL‐13 within the lung also induces CCL17 production by IRF4^+^ CD11b^+^ CD103^−^ DCs, required to recruit memory CD4^+^ T cells to the inflamed lung tissue.^68^ In the intestine, clustering of ILC3 with mononuclear phagocytes such as macrophages and DCs appears to support tolerance to dietary antigens, with macrophage‐derived IL‐1*β* stimulating ILC3 production of Csf‐2, which is then required to promote DC support for regulatory T cells.^33^, ^34^ In addition to effects from cytokines, cellular interactions between ILCs and antigen presenting cells (APCs) may also shape the adaptive response. For example NK cells can kill immature but not mature DCs and so NK cells can help to regulate the response through pruning of excess immature DC populations.^69^, ^70^

In addition to their effects on other haematopoietic cells, interactions between ILCs and local stroma may also influence B‐cell and T‐cell responses. In ontogeny the first known interactions of ILCs are with stromal cells, with a ROR*γ*t‐expressing ILC3 population termed lymphoid tissue inducer cells providing the critical source of lymphotoxin *α*_1_ *β*_2_ to stromal organizer populations and so stimulating local stromal cell differentiation, lymphoid tissue organogenesis and subsequent immune cell attraction.^57^ Marginal reticular cells comprise a subset of stromal cells within secondary lymphoid tissue that closely resemble, and are probably derived from, the embryonic stromal organizer population.^71^, ^72^, ^73^ Interestingly, marginal reticular cells are located in the same microenvironments where ROR*γ*t‐expressing ILC3 have been detected, which suggests continued interactions. In the spleen, such ILC3 interactions with marginal reticular cells are thought to contribute to marginal zone B‐cell antibody production.^52^ Further supporting ongoing interactions between stroma and ILCs, ILC3 contribute to splenic tissue remodelling after viral infection.^74^ Hence, it seems highly plausible that ILC3 interactions with stromal cells may influence stromal cell function and may contribute to the regulation of lymphocytes mediated by stroma within lymphoid tissues.^75^

### ILCs: regulation of lymphocytes through direct cellular interactions?

Further to the roles described above, direct cellular interactions between ILCs and lymphocytes are supported by several strands of evidence. ILC expression of MHCII, reported on both ILC2 and ILC3, suggests interactions specifically with CD4^+^ T cells and both these populations were able to process and present model antigens *in vitro*.^4^, ^5^ Among ILC3, expression of MHCII appears restricted to the CCR6^+^ population found predominantly in secondary lymphoid tissue and the gut (likely within the gut‐associated lymphoid tissue).^5^, ^42^ The signals governing MHCII expression on ILC3 are currently unclear, but appear to exclude common Toll‐like receptor signals associated with driving MHCII expression on classical APCs such as macrophages and DCs.^43^ Furthermore, ILC3 isolated from human small intestine also expressed MHCII.^5^ ILC2 show reduced expression of MHCII relative to ILC3 and classical APCs and its expression appears more site specific, perhaps reflecting as yet unknown environmental cues regulating its expression.^4^, ^5^

What do such interactions mean for CD4^+^ T‐cell responses? *In vitro* and *in vivo* data support MHCII‐dependent interactions between ILC2 and CD4^+^ T cells that enhance the resulting CD4^+^ T‐cell response.^4^, ^76^ A critical *in vivo* role for ILC3 in regulating the CD4^+^ T‐cell response to commensal bacteria was revealed by genetic deletion of MHCII on ILC3 (using ROR*γ*t‐cre × H2‐Ab1 floxed mice).^5^, ^43^ Interestingly, loss of MHCII expression on ILC3 *in vivo* (again using ROR*γ*t‐cre × H2‐Ab1 floxed mice) has also been shown to impair splenic CD4^+^ T‐cell responses following systemic immunization.^77^ Accepting that this might reflect differences in the microbiota of mice in distinct research facilities, these data suggest that the consequence of ILC3–CD4^+^ T‐cell interactions may depend on the type and/or site of infection. MHCII expression by ILC3 appears lower than that of DCs, suggesting that ILC3 may only efficiently present high‐affinity peptides to CD4^+^ T cells. It also remains to be determined how such peptides are acquired. Systemic memory CD4^+^ T‐cell responses were also substantially impaired in mice lacking ILC3; however, it is not known whether this effect is MHCII‐dependent.^40^

MHCII‐dependent associations obviously facilitate interactions between ILC3 and specific responding CD4^+^ T cells, but what mechanisms determine the outcome for the CD4^+^ T cell? ILC3 regulation of commensal T‐cell responses may be through sequestering available IL‐2.^43^ Although classical APCs are thought to provide key co‐stimulatory signals to T cells, clear evidence for co‐stimulatory molecule provision from ILCs is lacking. Notably ILCs appear to express a co‐stimulatory profile distinct from classical APCs and this is better suited to interactions with activated, rather than naive T cells.^78^ Studies of ILC3 report an absence^5^, ^79^ or very low levels of CD80 and CD86,^77^ but expression of tumour necrosis factor superfamily members such as lymphotoxin *α*_1_ *β*_2_, RANKL, OX40L, CD30L, LIGHT and other B7 family members such as ICOSL all provide potential mechanisms for ILC3 to contribute to sustaining the CD4^+^ T‐cell response after priming by more classical APCs.^53^, ^78^, ^79^, ^80^, ^81^, ^82^ ILC2 also show low or undetectable expression of CD80 and CD86,^4^ but do express ICOSL and also OX40L which may impact on interactions with CD4^+^ T cells.^83^, ^84^ One interpretation of these data is that ILC–CD4^+^ T‐cell interactions mediated by MHCII occur subsequent to those of naive CD4^+^ T cells, i.e. DC are the best cells at priming CD4^+^ T cells but ILC may then regulate the resulting population. This role is consistent with the location of these ILCs in secondary lymphoid tissue. The specific postnatal expression of OX40L and CD30L by murine ILC3,^79^, ^81^ is also suggestive of a change in ILC3 function after birth. Expression of many tumour necrosis factor superfamily members is tightly regulated and so specific provision of ligands at certain times during the response may occur. It is clear that for CD4^+^ T‐cell responses, signals through OX40 and CD30 are critical in sustaining a productive response.^85^, ^86^ Given that the co‐stimulatory interactions mentioned so far impact positively on CD4^+^ T‐cell responses, such data must be reconciled with the regulatory role described for ILC3 in T‐cell responses to commensal bacteria, and although effects on regulatory T cells are possible, none have been clearly demonstrated *in vivo* yet.^5^ Of course, it is possible that ILC provide co‐stimulatory molecules to CD4^+^ T cells in the absence of MHCII‐dependent interactions, as indicated for ILC2–Treg interactions in adipose tissue.^6^ In addition, site‐specific or response‐specific effects of ILCs on CD4^+^ T‐cell responses would seem entirely possible.

The role of ILCs in B‐cell responses remains poorly defined. There is evidence from human studies that splenic ILC3 support marginal zone B‐cell antibody production through effects on local cell populations and perhaps also through direct interactions.^52^ ILC3‐dependent support for CD4^+^ T‐cell responses will also impact on B‐cell help and there is some evidence for this in mice lacking MHCII on ILC3.^77^ Effects of ILC populations on CD8^+^ T‐cell responses have only been minimally explored and very recent studies suggested a possible role in regulating the homeostatic expansion of neonatal CD8^+^ T cells.^87^

## Conclusions and some outstanding questions

Given their relatively recent identification and the complexities of dissecting their contributions *in vivo*, it is hardly surprising that many questions remain concerning the importance of ILC populations, particularly with regard to adaptive immune responses. Sufficient evidence indicates that in certain situations ILC populations function to regulate the outcome of B‐cell and T‐cell responses. The challenge is to now robustly dissect the extent to which ILCs contribute to controlling adaptive immunity and the molecular interactions that underpin this. Armed with this knowledge, approaches to then try to therapeutically manipulate these pathways may reveal new ways to regulate immune responses. In the short‐term, experiments interrogating the following will provide key data to drive forward the possible translation of this research:

1. What are the T‐cell populations with which ILCs interact and where do interactions occur?
2. To what extent do ILCs process and present antigen *in vivo*? How is this antigen taken up and from what sources?
3. What is the extent of cross‐talk between stromal and ILC populations and what is the outcome of these interactions for both cell types?

## Disclosures

There is no conflict of interest.
